# Golestan cohort study of oesophageal cancer: feasibility and first results

**DOI:** 10.1038/sj.bjc.6602249

**Published:** 2004-12-14

**Authors:** A Pourshams, M Saadatian-Elahi, M Nouraie, A F Malekshah, N Rakhshani, R Salahi, A Yoonessi, S Semnani, F Islami, M Sotoudeh, S Fahimi, A R Sadjadi, D Nasrollahzadeh, K Aghcheli, F Kamangar, C C Abnet, F Saidi, V Sewram, P T Strickland, S M Dawsey, P Brennan, P Boffetta, R Malekzadeh

**Affiliations:** 1 Digestive Disease Research Center, Shariati Hospital, Tehran University of Medical Sciences, North Kargar Avenue, 14114 Tehran, Iran; 2 International Agency for Research on Cancer, Lyon, France; 3 Golestan University of Medical Sciences, Gorgan, Iran; 4 Cancer Prevention Studies Branch, Center for Cancer Research, National Cancer Institute, Bethesda, MD, USA; 5 PROMEC Unit, Medical Research Council, Tygerberg, South Africa; 6 Johns Hopkins Bloomberg School of Public Health, Baltimore, MD, USA

**Keywords:** oesophageal cancer, cohort, Golestan, turkmen, Iran

## Abstract

To investigate the incidence of oesophageal cancer (EC) in the Golestan province of North-East Iran, we invited 1349 rural and urban inhabitants of Golestan province aged 35–80 to undergo extensive lifestyle interviews and to provide biological samples. The interview was repeated on a subset of 130 participants to assess reliability of questionnaire and medical information. Temperature at which tea was consumed was measured on two occasions by 110 subjects. Samples of rice, wheat and sorghum were tested for fumonisin contamination. An active follow-up was carried out after 6 and 12 months. A total of 1057 subjects (610 women and 447 men) participated in this feasibility study (78.4% participation rate). Cigarette smoking, opium and alcohol use were reported by 163 (13.8%), 93 (8.8%) and 39 (3.7%) subjects, respectively. Tobacco smoking was correlated with urinary cotinine (*κ*=0.74). Most questionnaire data had *κ* >0.7 in repeat measurements; tea temperature measurement was reliable (*κ*=0.71). No fumonisins were detected in the samples analysed. During the follow-up six subjects were lost (0.6%), two subjects developed EC (one dead, one alive); in all, 13 subjects died (with cause of death known for 11, 84.6%). Conducting a cohort study in Golestan is feasible with reliable information obtained for suspected risk factors; participants can be followed up for EC incidence and mortality.

The earliest reports of high incidence of oesophageal cancer (EC) in the Northern parts of Iran date from the early 1970s (Kmet and Mahboubi, 1972). A population-based cancer registry was established in 1969, as a joint effort between Tehran University and the International Agency for Research on Cancer (IARC), in the city of Babol in Mazandaran province, on the Eastern side of the Caspian Littoral, and subsequently extended to the Western province of Gilan. This registry emphasised the high incidence of EC in the eastern portion close to Turkmenistan (Gonbad and Gorgan districts, now Golestan province), particularly in the semidesert plain settled mainly by people of Turkmen ethnicity, with incidence rates of 109 out of 100 000 among men and 174 out of 100 000 among women (Kmet and Mahboubi, 1972; Mahboubi *et al*, 1973). Sharp changes in the incidence of EC were evident in adjacent regions, being 15 among men and 5.5 out of 100 000 among women in Gilan, 300 km to the West. A series of studies in the 1970s were prematurely stopped in 1978, and though not conclusive in explaining the increased risk, pointed to several factors including: (i) a diet deficient in fruits and vegetables (Cook-Mozaffari *et al*, 1979); (ii) a thermal injury from consumption of very hot beverages (Cook-Mozaffari *et al*, 1979; Ghadirian, 1987); and (iii) carcinogen exposure from lifestyle factors including opium consumption (Joint Iran-IARC Study Group, 1977; Dowlatshahi and Miller, 1985; Ghadirian *et al*, 1985). Aetiological hypotheses related to diet, including temperature of beverages (Munoz and Day, 1996), exposure to mycotoxins such as fumonisins (Wang *et al*, 2000; Abnet *et al*, 2001a; Shephard *et al*, 2002), and use of illicit products can be best addressed in prospective studies, in which measurement error is reduced and recall bias is absent (White *et al*, 1998). To reopen the investigation, an initiative involving the Digestive Diseases Research Centre (DDRC) of Tehran University of Medical Sciences, the IARC, and the US National Cancer Institute (NCI) has therefore been established, aiming to conduct a cohort study in this region. Such a prospective cohort study will include at least 50 000 individuals, and requires a carefully planned feasibility phase. Here we present the details of the feasibility study and initial results on lifestyle factors, including tea temperature.

## METHODS

A protocol for the pilot study was developed jointly by research teams from the DDRC, IARC and NCI with the aim (i) to evaluate the logistical aspects of the project, including the response rate of the study population, the acceptability of the questionnaires, and the procedures for collecting and storing biological samples; (ii) to assess the reliability of interviews by conducting repeated interviews on a sample of subjects; (iii) to validate questionnaire-based opium consumption data with biological markers of opium consumption measured in urine and hair samples. The protocol concentrated on identifying a methodology for recruiting subjects, developing a lifestyle questionnaires and a nutritional component, collecting information on anthropometric measures, assessing the maximum temperature at which an individual could comfortably drink tea, and collecting biological samples. The main questionnaire was developed in order to obtain information on demographic and socioeconomic details as well as past and present medical history, types of fuel used for heating and cooking, history of cancer in the relatives, gastrointestinal symptoms, and body shape at age 15, age 30 and present age. Information on recent and past consumption of alcohol, cigarettes, nass (a smokeless tobacco product containing also ash, lime and oil) and opium was collected. In a subset of 130 subjects, a detailed semiquantitative food-frequency questionnaire (FFQ) was administered four times and 12 24-h dietary recalls for two consecutive days administered monthly during 1 year. The urinary excretion of nitrogen was estimated from four 24-h urine collection of 118 subjects for determination of protein intake by Isakson formula, and plasma levels of *β*-carotene, retinol, vitamin C and vitamin E from two nonfasting blood collections in 125 subjects.

Subjects for the pilot study were selected during the summer of 2002 from Gonbad, the second biggest city of the province, and three rural villages in the surrounding region in Golestan province (Incheborun, Hali-Akhond and Aq-Abad). Recruitment took place at the Golestan Cohort Study Centre (GCSC) in Gonbad, a research centre specifically established for this project, and in the health house of each village. Health houses are present in each village and staffed by two auxiliary health personnel (Behvarz), who are in charge of vaccination programs, family planning, report of death and major communicable diseases and initial primary care treatment. In the villages, trained Behvarz selected households by systematic clustering according to household number, contacted all household members aged between 35 and 80 (*N*=704) and thoroughly explained them the purpose and procedure of the study. Residents in Gonbad (*N*=645) were selected by household sampling and contacted at their home by expert local health professionals, who thoroughly explained them the purpose and procedure of the study. Individuals were invited to participate by attending the health house or the GCSC at a specific time for interview. Interviewers were local physicians and, in the case of dietary interviews, nutritionists who were trained for this purpose.

Exclusion criteria used in this study were: (i) unwillingness to participate at any stage of the study for any reason; (ii) being a temporary resident; and (iii) having had a diagnosis of upper gastrointestinal cancer. Before interview, a written informed consent was obtained. Support from local leaders was also obtained.

The interview was conducted by a trained general physician either in the local language (Turkmen) or in Persian, depending on the subject's preference. After the interview, a limited physical examination was performed including the measurement of height, weight, number of lost teeth and blood pressure. A repeated interview was performed on 130 subjects – 50 urban and 80 rural – 2 months after the first interview.

In order to measure temperature of tea drinking, subjects were offered during the interview a fresh cup or glass of tea. A second cup was kept by the interviewer, who measured the temperature with an alcohol thermometer at the moment in which the subject drank his/her tea. Since the analysis of repeated measurements showed poor reliability of this method (*κ* 0.09, based on 130 repeats), an alternative approach was tested on 110 randomly selected participants, who were re-contacted. In this case, two measurements were made at the home of the subjects one day apart. The health worker prepared a fresh cup of tea and measured the temperature using a digital thermometer. When the tea was at 75°C, subjects were asked if they could drink the tea. If this was not possible, the tea was let to cool to 70°C, and subjects were asked again if they could drink the tea. This procedure was repeated, at 5°C intervals, until it was possible for the subjects to drink the tea.

Blood samples (10 ml) were collected in the health houses, kept in a freezer box and transported to the GCSC every evening, where they were separated into buffy coat, plasma and red blood cells and stored in colour-coded 1.8-ml tubes at −80°C. Subjects were also asked to provide a hair sample, taken from the nape of the neck, as well as a nail sample and a urine sample.

Hair and nail samples were stored at room temperature and urine samples at −20°C. In order to assess the validity of self-reported tobacco smoking, urine samples from 96 participants were selected randomly and sent to the Johns Hopkins University, Baltimore, MD, USA, where they were measured for cotinine using NicoMeter® strips.

Samples (100 g) of raw rice, sorghum and wheat used for bread production were collected from selected households included in the feasibility study. They were kept at −10°C until they were sent to the Medical Research Council, Tygerberg, South Africa, for fumonisin analysis. The dry grain samples were ground to a meal and analysed for the presence of fumonisins B_1_, B_2_ and B_3_ using the method of Sydenham *et al* (1996). In brief, ground samples (20 g) were homogenised in 70% aqueous methanol (100 ml) for 3 min. Aliquots of the filtered extracts (10 ml) were cleaned up on strong anion exchange (SAX) solid phase extraction (SPE) cartridges (Bond Elut LRC®) that were conditioned with methanol (5 ml) and 70% aqueous methanol (5 ml). The extract was eluted with 1% acetic acid in methanol and evaporated to dryness at 60°C under a continuous flow of nitrogen. Residues were redissolved in methanol, and an aliquot was derivatised with *o*-phthaldialdehyde prior to separation on a reversed phase HPLC system using fluorescence detection.

Study participants were contacted 6 and 12 months after recruitment in rural areas by the Behvarz and in urban areas by health professionals. Information on date and cause of death was collected for those who died, as was information of incidence of EC. In addition, local hospitals, including in particular the Atrak Clinic, a hospital based Clinic specialised in gastrointestinal diseases and established in Gonbad by DDRC, were contacted to identify further cases of EC. Subjects included in the study were advised to refer to the Atrak Clinic for symptoms possibly associated with digestive diseases.

The study protocol and the informed consent used for this investigation were approved by the ethical review committees of DDRC and the IARC. The analysis of data and samples was approved by ethical review committee of the NCI.

## RESULTS

In Gonbad, 438 subjects of the 645 who we invited visited the GCSC for interview and provided biological samples (participation rate 67.9%); in the three rural villages, 619 individuals participated successfully (participation rate 87.9%). The higher response rate among the rural inhabitants is explained by their employment close to home in agricultural occupations, and their flexible working hours and proximity of their homes with work place and health houses. In total, 1057 subjects were enrolled into the feasibility study, including 447 men and 610 women (overall participation rate, 78.4%). Selected demographic characteristics of the study population are reported in Table 1. Results of the analysis for use of alcohol, tobacco and opium are shown in Table 2. Alcohol drinking was restricted to men and was rarely reported in the rural areas. Tobacco smoking was mainly reported by men. Consumption of nass and opium was particularly common among rural men, although some rural women admitted opium consumption. Urinary cotinine was positive in 46% of the study subjects; there was a good agreement between urinary cotinine positivity and self-reported current tobacco smoking or nass use (Pearson correlation coefficient=0.73, *P*<0.0001).

Symptoms related to gastro-oesophageal reflux disease were commonly reported among both rural and urban participants (Table 3). Recent regurgitation was reported by 49.8% of rural and 48.6% of urban participants. The prevalence of self-reported heart diseases and diabetes was significantly higher among urban subjects, and that of chronic connective tissue and joint diseases was higher among rural subjects. The prevalence of other chronic diseases was comparable in the two groups. The prevalence of overweight and obesity was high, in particular among urban participants. The average total number of teeth was 15 (s.d.±10) among rural and 16 (s.d.±11) among urban participants.

The analysis of reliability of questionnaire information, based on repeated interview on 130 subjects, showed that *κ* values were above 0.7 for most variables, including tobacco, nass, opium and alcohol consumption, as well as for most self-reported symptoms and for anthropometric measures (Table 4). Measurement of HBsAg, blood group and Rh group was performed on repeated blood samples, with 100% agreement.

The detailed results of the repeated measurements of tea temperature are shown in Table 5. The level of agreement between the two separate measurements of tea temperature was good (weighted *κ*=0.71) with most disagreements occurring in adjacent categories. Among the six individuals who reported being able to drink tea in the highest temperature category (>70°C), five of them gave the same response at the second measurement, whereas the sixth reported drinking tea in the 66–70°C range.

The analysis of 10 grain samples revealed no fumonisin contamination and following these preliminary results, no further analyses were performed.

There was good correlation based on food groups and nutrients comparing 12 recalls to four FFQ questionnaires. There was acceptable correlation between questionnaire data and biomarker measurement except for vitamin E.

The detailed data on dietary and nutritional variables is in the process of a separate publication, but overall there were significant difference in consumption of some food group and nutrients between Persians and Turkmen. Persians consume significantly more legumes, fruits, vegetables while Turkmen consume more nonalcoholic beverage (tea). Protein intake in Persian woman was more than Turkmen woman.

Results on opium exposure have been reported previously (Abnet *et al*, 2004); results on dietary factors, including nutrient level, will be the subject of a separate report.

The results of the follow-up are summarised in Table 6. One study participant was lost to follow-up (0.1%) and five subjects emigrated from the province (0.5%). Information on cause of death was available from 11 out of 13 subjects who died during the first year of follow-up (84.6%). Six of these 11 deaths were due to cardiovascular diseases, two to car accident, one subject died from breast cancer and another one from pneumonia. Two cases of EC were identified, one of whom died from the disease.

## DISCUSSION

The pilot study has demonstrated the feasibility of recruiting fairly large numbers of adult subjects from urban and rural areas of the Golestan province with a high response rate, and of obtaining both interview information and biological samples. The high participation rate and the successful follow-up in rural areas were due to the excellent health infrastructure with 95% coverage in the study area, the close relationship between Behvarz and inhabitants of the area and the support obtained from local leaders.

Repeat interviews on a subsample confirmed the reliability of most questionnaire data. The proposed method for obtaining information on tea temperature was also reliable. The pilot study also demonstrated that there exists significant between-subject variation for all of the main exposures of interest, and in particular tea temperature and opium consumption, which will increase our ability to detect their aetiological role in EC.

Prevalence of adenocarcinoma of the oesophagus in Gonbad is still low (Islami *et al*, 2004). However, the high prevalence of reflux-like symptoms in Gonbad, as compared to Asia (Ho *et al*, 1998), overweight and obesity suggest that an increasing incidence of adenocarcinoma of distal oesophagus may be observed in the future in this population. The low number of teeth suggests that the subjects have poor oral hygiene and associated oral bacteria may have a role in the aetiology of EC in this population. Previous reports have shown that poor oral hygiene is associated with increased production of carcinogenic nitrosamines (Nair *et al*, 1992) and tooth loss has been associated with increased risk of oesophageal squamous cell and gastric cancers in another high-risk population (Abnet *et al*, 2001b). The high prevalence of self-reported ischaemic heart disease, diabetes and hypertension is consistent with our finding of high mortality from cardiovascular diseases during the first year of follow-up.

Our pilot study confirms previous findings of a low prevalence of tobacco smoking, nass use and alcohol drinking in this population, particularly among women (Joint Iran-IARC Study Group, 1977). These factors are therefore probably not major aetiological factors for EC in this area (Islami *et al*, 2004). Although, mycotoxin contamination of cereals does not seem to play an important aetiological role, our analyses were based on a limited number of samples, and any conclusion on the possible role of fumonisins in this population is premature. We plan to conduct further analyses (i) to characterise the contamination of cereals by different fungal groups and (ii) to measure fumonisins in hair samples of study subjects. Opium use, on the other hand, remains a fairly common habit, particularly among rural men of Turkmen ethnicity. Previous studies from north Iran suggested a role of opium metabolites in oesophageal carcinogenesis (Hewer *et al*, 1978; Malaveille *et al*, 1982; Friesen *et al*, 1985; 1987; Ribeiro Pinto and Swann, 1997).

In total, 51 of the subjects included in the validation study (46.4%) drank tea on two occasions at a temperature of 65°C or higher. This strongly suggests that high tea temperature is a factor in EC in this population. A study from the 1970s showed 62% of Iranians in the high-risk region, as opposed to 19% in the low-risk region, used to drink their tea at a temperature above 65°C (Ghadirian, 1987). Furthermore, inhabitants of high-risk areas consumed 2.5 times more tea than their counterparts in the low-risk areas. A role of drinking of hot beverages, such as tea and maté, has been reported in studies conducted in different regions, including United Kingdom (Sharp *et al*, 2001), South America (Castellsague *et al*, 2000; Sewram *et al*, 2003) and Turkey (Onuk *et al*, 2002).

This Pilot study also successfully validated the reliability of our semiquantitive FFQ for food groups, energy and nutrients (macronutrients, retinol, vitamins C and E, *β*-carotene), all considered to be important in EC aetiology. We would therefore be able to use this reliable FFQ in measurements of food intake for all nutrient groups in our main cohort study.

The short-term follow-up required for this pilot study was found to be straightforward and feasible, using information from a combination of sources including Behvarz in rural areas, health professionals in Gonbad, local hospitals, Atrak Clinic, and the ongoing Golestan cancer registry. We intend to rely on these sources for long-term follow-up in the actual cohort and will also add an active follow-up team of three trained interviewers to contact all urban study subjects once each year.

From the total annual number of cases seen at Atrak Clinic and the number of the reported cases to the ongoing cancer registry, we expect that 400 EC cases will be observed in the first 5 years of the actual cohort study.

In conclusion, a cohort study is feasible in Northern Iran, allowing relevant suspected aetiological factors to be investigated both in terms of prevalence of exposure and ability to obtain reliable and valid measurements. Our experience may be relevant to other developing countries, in which the health system is based on local health centres covering the whole population, local expertise in epidemiological research is available, and the project has local support.

## Figures and Tables

**Table 1:** Selected characteristics of study population, by residence

	**Rural (*N*=619)**		**Urban (*N*=438)**	
	***N***	**%**	***N***	**%**
*Age group (year)*				
<40	99	15.9	75	17.1
40–49	216	34.9	154	35.2
50–59	159	25.8	98	22.4
60–69	80	12.9	68	15.5
⩾70	65	10.5	43	9.8
				
*Sex*				
Male	270	43.6	177	40.4
Female	349	56.4	261	59.6
				
*Ethnicity*				
Turkmen	599	96.8	65	14.8
Persian	3	0.5	285	65.1
Turk	0	—	68	15.5
Other	17	2.7	20	4.6
				
*Education level*				
Illiterate	464	74.9	115	26.3
Primary school	126	20.4	144	32.9
Guidance	7	1.1	32	7.3
High school	8	1.3	80	18.3
College	6	1	58	13.2

**Table 2:** Use of alcohol, tobacco and opium, by sex and residence

**Exposure**	**Rural men, *N* (%)**	**Urban men, *N* (%)**	***P*-value^a^**	**Rural women, *N* (%)**	**Urban women, *N* (%)**	***P*-value^a^**
*Alcohol drinking*						
Ever	8 (3.0)	31 (17.3)	<0.0001	0 (0.0)	0 (0.0)	—
Frequency^b^						
<50 g day^−1^	1 (12.5)	20 (66.7)	0.02	—	—	—
50–150 g day^−1^	3 (37.5)	4 (13.3)		—	—	
>150 g day^−1^	4 (50.0)	6 (20.0)		—	—	
						
*Tobacco smoking*						
Ever	81 (30.0)	69 (38.5)	0.06	4 (1.1)	9 (3.4)	0.04
Recent	53 (19.6)	42 (23.5)	0.3	1 (0.3)	5 (1.9)	0.009
Frequency (cpd)^b^						
<2	3 (3.9)	3 (4.6)	0.6	2 (50.0)	2 (28.6)	0.6
2–3	54 (71.1)	50 (76.9)		2 (50.0)	4 (57.1)	
4–6	19 (25.0)	12 (18.5)		0 (0.0)	1 (14.3)	
						
*Nass use*						
Ever	50 (18.5)	3 (1.7)	<0.0001	2 (0.6)	0 (0.0)	0.22
Recent	47 (17.4)	1 (0.6)	<0.0001	2 (0.6)	—	—
Frequency^b^						
<1 day^−1^	2 (4.1)	0 (0.0)	0.8	0 (0.0)	—	—
1–5 day^−1^	17 (34.7)	1 (33.3)		2 (100.0)	—	
6–20 day^−1^	20 (40.8)	2 (66.7)		0 (0.0)	—	
>20 day^−1^	10 (20.4)	0 (0.0)			—	
						
*Opium use*						
Ever	58 (21.5)	14 (7.9)	<0.0001	19 (5.1)	2 (0.8)	0.03
Recent	53 (19.6)	9 (4.5)	<0.0001	15 (4.3)	1 (0.4)	0.04
Frequency^b^						
<1 day^−1^	12 (23.1)	4 (28.6)	0.2	3 (18.7)	0 (0.0)	0.8
1 day^−1^	14 (26.9)	7 (50.0)		6 (37.5)	1 (50.0)	
2–5 day^−1^	22 (42.3)	3 (21.4)		7 (43.7)	1 (50.0)	
6+ per day	4 (7.7)	0 (0.0)		0 (0.0)	0 (0.0)	

a *P*-value of difference between rural and urban populations (*χ*^2^ test, two-sided).

b Percentages refer to ever users cpd, cigarettes per day.

Numbers might not add up because of missing values.

**Table 3:** Prevalence of gastroesophageal reflux disease (GERD), chronic diseases and body mass index, by residence

	**Rural, *N*=619**	**Urban, *N*=438**	
	***N* (%)**	***N* (%)**	***P*-value^a^**
*Any GERD symptom* b			
Never	316 (51.0)	219 (50.0)	0.7
1–6 times per week	110 (17.7)	74 (16.9)	
Daily	82 (13.2)	66 (15.1)	
			
*Heartburn*			
Never	349 (56.3)	287 (65.5)	0.2
1–6 times per week	88 (14.2)	49 (11.2)	
Daily	51 (8.2)	45 (10.3)	
			
*Regurgitation*			
Never	318 (51.4)	219 (50.0)	0.6
1–6 times per week	101 (16.2)	66 (15.1)	
Daily	66 (10.6)	54 (12.3)	
*History of chronic diseases* (*ever)*			
Any diseases	169 (27.3)	158 (36.1)	0.002
Heart diseases	7 (1.1)	30 (6.8)	<0.0001
Stroke	7 (1.1)	7 (1.6)	0.5
Hypertension	81 (13.1)	71 (16.2)	0.1
Diabetes	17 (2.7)	35 (8.0)	0.0001
Pulmonary diseases	16 (2.6)	11 (2.5)	0.9
Connective tissue and joint disease	38 (6.1)	4 (0.9)	<0.0001
Renal failure	1 (0.2)	0 (0.0)	0.4
			
*BMI (kg m*^*−2*^)			
<18.5	55 (8.9)	3 (0.7)	<0.0001
18.5–24.9	284 (45.7)	109 (24.6)	
25.0–29.9	177 (28.5)	168 (38.0)	
30.0+	107 (17.2)	163 (36.7)	

a *P*-value of difference between rural and urban populations (*χ*^2^ test, two-sided).

b Heartburn or regurgitation.

**Table 4:** Repeatability of selected characteristics

**Characteristic**	**Agreement (%)**	***κ***
Gender	97.8	0.95
Residence place	100.0	1.00
Age	100.0	1.00
Ethnicity	99.3	0.98
Marital status	99.3	0.96
Education level	96.3	0.94
Working at present	91.9	0.62
Working start age	67.1	0.53
Cancer in relatives	97.1	0.92
Alcohol drinking status	99.3	0.93
Cigarette smoking status	99.3	0.97
Cigarette smoking (amount)	100.0	1.00
Nass use status	99.3	0.95
Nass use (amount)	70.0	0.58
Opium use status	99.3	0.96
Opium use (amount)	81.8	0.73
Heartburn (frequency)	68.9	0.60
Regurgitation (frequency)	84.4	0.79
Vomiting (frequency)	85.7	0.72
Teeth count	88.3	0.86
Ischaemic heart disease	96.4	0.78
Stroke	100.0	1.00
Hypertension	96.4	0.83
Diabetes mellitus	99.3	0.85
Water supply	100.0	1.00
Income	55.6	0.39
House surface (m^2^)	71.4	0.59
Height	85.2	0.83
Weight	89.3	0.86
Body mass index	82.3	0.72

**Table 5:** Repeatability of measurement of tea temperature

	**Second day**					
**First day**	**<60**	**60**	**65**	**70**	**>70**	**Total 1st**
<60	1	4	0	0	0	5
60	3	24	10	1	0	38
65	1	13	23	3	0	40
70	0	0	13	6	0	21
>70	0	0	0	1	5	6
Total 2nd	5	41	46	13	5	110

**Table 6:** Results of the follow-up

**Outcome**	**1–6 months**	**7–12 months**	**Total**
Loss-to-follow-up	1	0	1
Emigration	1	4	5
Deaths	7	6	13
Known cause of death	6	5	11
Cases of oesophageal cancer	0	2^a^	2^a^

a Including one alive case.
